# Biodistribution and Molecular Studies on Orally Administered Nanoparticle-AON Complexes Encapsulated with Alginate Aiming at Inducing Dystrophin Rescue in *mdx* Mice

**DOI:** 10.1155/2013/527418

**Published:** 2013-12-12

**Authors:** Maria Sofia Falzarano, Chiara Passarelli, Elena Bassi, Marina Fabris, Daniela Perrone, Patrizia Sabatelli, Nadir M. Maraldi, Silvia Donà, Rita Selvatici, Paolo Bonaldo, Katia Sparnacci, Michele Laus, Paola Braghetta, Paola Rimessi, Alessandra Ferlini

**Affiliations:** ^1^Department of Medical Sciences, University of Ferrara, 44121 Ferrara, Italy; ^2^Unit of Molecular Medicine for Neuromuscular and Neurodegenerative Diseases, Bambino Gesù Children's Hospital, IRCCS, 00146 Rome, Italy; ^3^Department of Biology and Evolution, University of Ferrara, 44121 Ferrara, Italy; ^4^IGM-CNR, Unit of Bologna c/o IOR, 40136 Bologna, Italy; ^5^Department of Biomedical Sciences, University of Padua, 35121 Padua, Italy; ^6^Department of Environmental and Life Sciences INSTM, University of Eastern Piedmont, 15121 Alessandria, Italy; ^7^Department of Medical Sciences, Section of Medical Genetics, University of Ferrara, via Fossato di Mortara 74, 44121 Ferrara, Italy

## Abstract

We have previously demonstrated that intraperitoneal injections of 2′-O-methyl-phosphorothioate (2′OMePS) antisense oligoribonucleotides adsorbed onto a cationic core-shell nanoparticles (NPs), termed ZM2, provoke dystrophin restoration in the muscles of *mdx* mice. The aim of the present work was to evaluate the oral route as an alternative way of administration for ZM2-antisense oligoribonucleotides complexes. The biodistribution and elimination of nanoparticles were evaluated after single and multiple oral doses of IR-dye conjugated nanoparticles. Labeled nanoparticles were tracked *in vivo* as well as in tissue cryosections, urines and feces by Odyssey infrared imaging system, and revealed a permanence in the intestine and abdominal lymph nodes for 72 hours to 7 days before being eliminated. We subsequently tested alginate-free and alginate-encapsulated ZM2-antisense oligoribonucleotides (AON) complexes orally administered 2 and 3 times per week, respectively, in *mdx* mice for a total of 12 weeks. Treatment with alginate ZM2-AON induced a slight dystrophin rescue in diaphragm and intestine smooth muscles, while no dystrophin was detected in alginate-free ZM2-AON treated mice. These data encourage further experiments on oral administration testing of NP and AON complexes, possibly translatable in oligoribonucleotides-mediated molecular therapies.

## 1. Introduction

The X-linked recessive Duchenne muscular dystrophy (DMD) affects 1 in 3500 newborn boys [1], and it is caused by the loss of dystrophin expression. The antisense mediated exon skipping approach represents a promising therapy for DMD. It is based on the possibility to convert a severe phenotype (DMD) into a milder form (Becker muscular dystrophy) acting on dystrophin pre-mRNA [2, 3].

Two AON chemistries, 2′OMePS and phosphorodiamidate morpholino oligomer (PMO), have already been the subject of clinical trials in humans [1, 4–8]. Phosphorothioate (PS) oligonucleotides are the most widely studied AONs due to their stability to nucleolytic degradation and relative ease of synthesis. In PS oligonucleotides, the nonbridging oxygen is replaced with a sulfur atom in the oligonucleotide chain. This substitution confers sufficient stability in plasma, tissues, and cells to avoid degradation before binding to target RNA. PS-modified AONs are water-soluble and have high protein-binding capacity, which prevents rapid renal excretion and facilitate uptake into tissues. PMO consists of the replacement of the phosphodiester bond by a phosphorodiamidate linkage with the ribose replaced by a morpholino moiety. PMOs are charge neutral, refractory to biological degradation and stable in serum and plasma [9–11].

**Figure 1:**
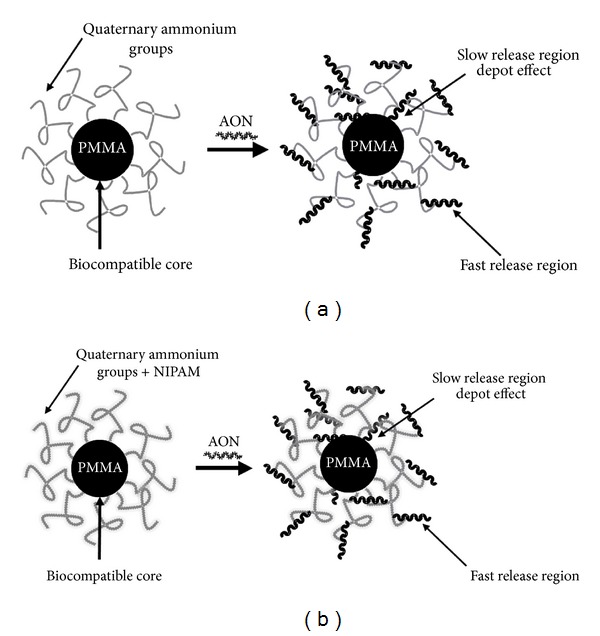
Nanoparticles characteristics. Representation of the interactions between antisense oligoribonucleotide (M23D AON) and quaternary ammonium groups on the surface of T1 (a) and ZM2 (b) nanoparticles.

In order to increase stability and half-life in biological fluids, thereby improving AON efficacy, different polymeric nanoparticles have been developed; their subcellular and submicron size allows them to penetrate deeply into tissues through the fine capillaries, crossing the fenestration present in the epithelial lining. We have previously demonstrated the effectiveness of polymethylmethacrylate (PMMA) T1 (Figure 1(a)) nanoparticles conjugated with AON to induce dystrophin restoration in body-wide muscles in the *mdx* animal model. Although we obtained functional effect with one-eightieth of the normal AON dose regimen, the efficiency of this compound was relatively unsatisfactory in terms of size, AON loading capacity, and efficiency of treatment. Therefore, to obtain a more efficient compound, we designed and prepared a novel type of cationic core-shell NP (termed ZM2), made up of a predominantly PMMA core and a random copolymer shell consisting of units derived from N-isopropyl-acrylamide+ (NIPAM) and reactive methacrylate-bearing cationic groups (Figure 1(b)). ZM2 nanoparticles were able to bind and deliver 2′OMePS M23D AON in *mdx* mice, inducing dystrophin restoration in the skeletal muscles, arrector pili smooth muscle, and heart after intraperitoneal (i.p.) treatment [12–15].

The *mdx* mouse is the most widely used animal model for exon skipping experiments since it is a natural mutant with a stop mutation within exon 23 of the *dystrophin* gene. This causes a complete absence of dystrophin protein as well as a moderate muscle weakness. This stop mutation can be corrected using the exon skipping approach [16].

Indeed, treatment of *mdx* mice with 2′OMePS M23D AON induces the exclusion of the mutated exon 23 from mRNA resulting in an in-frame mRNA transcript and subsequent expression of a slightly shorter dystrophin protein in *mdx* muscle [12, 17–19].

The objective of this work was to study the biodistribution, elimination, and efficacy of orally administered NPs-AON in *mdx* mice. The oral route is considered the most attractive for small molecules and macromolecular drug delivery, encompassing the advantages of easy administration, high patient compliance, and cost-effectiveness [20, 21]. The PS oligonucleotides molecules are poorly bioavailable when administered orally for their low mucosal permeability and poor transcytosis across the gut, due to both unfavorable physicochemical properties (hydrophilicity, high molecular weight, high negative charge density) and to the depurination in the acidic gastric environment [22–25].

In order to protect molecules and facilitate drug permeation, different kinds of delivery systems are under study in order to inhibit hydrolysis, prolong intestinal retention, and thereby enhance drug bioavailability [26–30].

Alginate is one of the most common and suitable biopolymers. It is nontoxic, biodegradable, and employed to make acidic pH-resistant hydrogel [31]. The most important property of alginates is their ability to form a reticulated structure in the presence of calcium ions, allowing the entrapment of molecules, making them good mucoadhesive agents [32, 33]. Alginate is a family of polysaccharides composed of *α*-L-guluronic acid (G) and *β*-D-mannuronic acid residues (M), arranged in homopolymeric blocks of each type and in heteropolymeric blocks [31].

Swelling of alginate is minimal in the stomach, while it increases moving toward the intestine, due to the pH increase [34]. In the present paper, we describe the biodistribution and elimination of ZM4 NPs conjugated with the dye NIR-797 after single and multiple oral administrations, using the Near-Infrared fluorescent imaging System Odyssey (LI-COR Biosciences). The biodistribution studies indicate that NPs persist in the intestinal lumen for at least 72 hours after a single administration and are concentrated in the intestine and abdominal lymph nodes after multiple administrations before being completely eliminated at day 7 from the last treatment. Successively, we report on the results obtained using alginate as encapsulating agent for ZM2-AON complexes orally administered in *mdx* mice. *mdx* mice orally treated with alginate-free ZM2-M23D or alginate-coated ZM2-M23D revealed a slight rescue of dystrophin protein in the intestinal smooth muscles and a mild positivity in the diaphragm only when in the presence of alginate. The low rescue was associated with the absence of AON in tissues, as assayed by AON specific ELISA.

These results encourage further research into the oral administration route for antisense molecules.

## 2. Results

### 2.1. Biodistribution and Elimination of Nanoparticle Oral Treatment

To assess the biodistribution and elimination pathways, a novel NP sample was prepared, nominated ZM4. ZM4 features the same size, size distribution, and surface hydrophilicity as ZM2, but it also exposes at the surface, or in the swelled shell, primary amino groups. These groups are necessary to interact with the isothiocyanate groups of the NIR-797 fluorescent dye (Sigma-Aldrich), giving rise to ZM4-IR nanoparticles in which the dye is covalently incorporated into the shell (see Table 1 reporting the NPs characteristics). The *in vivo* biodistribution was evaluated, after a single or multiple oral administrations, employing the Odyssey Imaging System (Li-Cor Biosciences) that allows the specific distribution and clearance of the infrared dye-labeled nanoparticles to be tracked in live animals over time. See Table 2 for the schedule of treatments.

**Figure 2:**
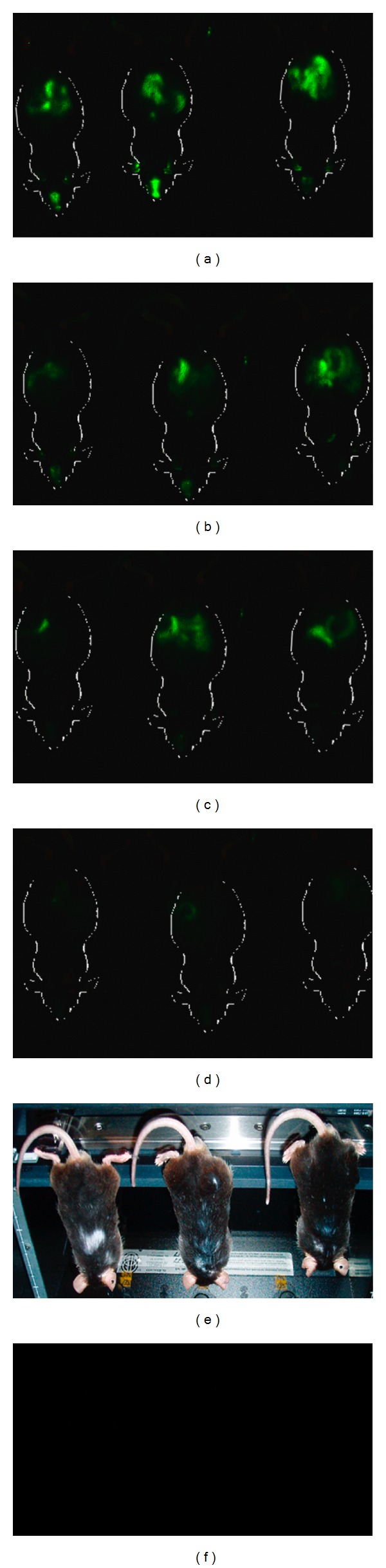
Biodistribution of ZM4-IR-dye. Three mice (1, 2, 3) were analyzed at 12 (a), 24 (b), 48 (c), and 72 (d) hours post ZM4-IR single administration. Fluorescence was visualized using the Odyssey Infrared Imaging System. Green fluorescence represents the IR signal (~800 nm) associated to the nanoparticle. The signal appears localized to the abdominal region (intestine) of the mouse. (e) Mice located on the mouse pod of the Odyssey scanner. (f) The image shows the absence of fluorescence signal in *mdx* untreated mice.

The Odyssey *in vivo* imaging time course of ZM4-IR after single or multiple administrations demonstrated the persistence of ZM4-IR in the intestinal lumen for at least 72 hours (Figure 2), and the absence of any NP accumulation. The positivity observed in the mouth (masseter) is due to residues of NP IR-dye formulations administered by gavage.

**Figure 3:**
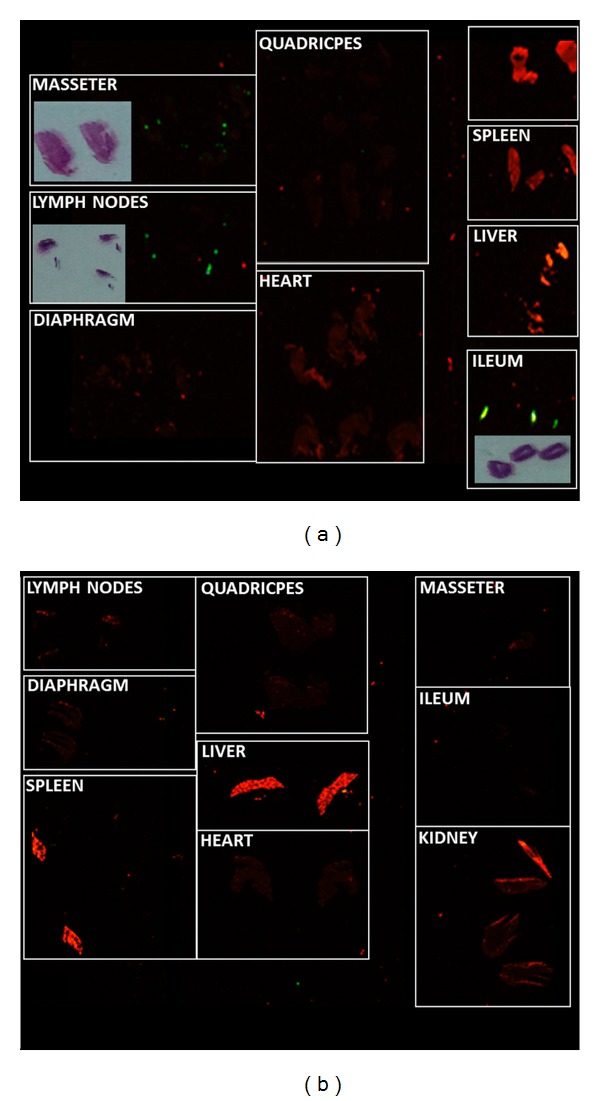
Odyssey analysis of organ/muscle cryosections (20 *μ*m) from ZM4-IR multiple-dose administered mice 7 days (a) and 1 month (b) after last treatment. Green fluorescence represents IR-dye signal (~800 nm) associated to the nanoparticle; red represents tissue autofluorescence at ~700 nm. The signal is evident in abdominal lymph nodes, ileum, and masseter muscle at 7 days and absent at 1 month. Hematoxylin-Eosin (HE) staining of the same cryosections is presented next to IR positive sections to clarify the position on the slide.

In the multiple administration schedule, ZM4-IR treated *mdx* mice were killed following one week (*n* = 3) or one month (*n* = 3) from administration of the last dose. The analysis of cryosections from different organs shows that ZM4-IR NPs are still present in intestine and abdominal lymph nodes (Figure 3(a)) one week after treatment, while no NPs were detected in the organs of mice sacrificed 1 month after the last administration (Figure 3(b)). The positivity of the masseter muscle is probably due to local adsorption as a result of oral administration by gavage.

In the nanoparticles clearance studies, feces and urine samples were collected and analyzed with Odyssey. The feces of 3 untreated *mdx* mice were used as negative controls. Neither the feces nor urine samples of the untreated mice showed any fluorescence, while the feces of treated mice showed an intense signal. NP clearance occurred almost exclusively through the feces and mainly in the first 48 hours after administration. In fact, by semiquantitative analysis it resulted that 90% of administered nanoparticles was detected in the feces samples collected during the first 12–36 hours after treatment, while the remaining 10% was gradually eliminated in the next few days, being completely removed after 7 days (data not shown).

### 2.2. Dystrophin Restoration Studies: ZM2-M23D Complexes

#### 2.2.1. *mdx* Mice Treatment

Table 3 summarizes the oral treatments and the sacrifice of mice analyzed in this work: 3 *mdx* mice were administered for 12 weeks (2 doses/week) with ZM2-M23D complexes; 3 *mdx* mice were treated for 12 weeks with AON-free ZM2; 3 *mdx* mice were treated for 12 weeks (3 doses/week) with ZM2-M23D complexes coated with alginate; 3 *mdx* mice were treated for 12 weeks with alginate coated AON-free ZM2; 3 untreated mice were included as negative controls.

The amount of AON loaded onto alginate-coated ZM2 nanoparticles was lower with respect to the one used for ZM2 uncoated nanoparticles, 200 *μ*g versus 225 *μ*g, in order to facilitate the interaction of the free surface positive charges of ZM2-AON complexes with alginate. For this reason the number of injections/week (2 versus 3) are different. However the total amount of M23D oligonucleotide received in each group of treatment was the same, represented by 240 mg/kg. All groups of mice were sacrificed 1 week after the last administration.

The only side effect we observed was a mild laxative effect after the treatment with alginate formulations.

#### 2.2.2. RNA Analysis

In order to test the effect of ZM2-AON treatments on the dystrophin transcript, we assessed exon 23 skipping levels using a Real-Time PCR exon-specific assay (ESRA), as previously reported [13, 35]. ESRAs were performed using the RNA pool for each treatment group and skipping percentages were calculated for the treated compared to the untreated mice. After 12 weeks of treatment with alginate-ZM2-AON, we only detected a low level of exon 23 skipping in the diaphragm of mice treated with alginate-coated ZM2-AON (mean skipping value for 3 mice, 8%). Exon-23 skipping was undetectable in the cardiac muscle, gastrocnemius, quadriceps and intestine for both ZM2-AON and alginate-ZM2-AON treated *mdx* mice.

#### 2.2.3. AON Hybridization Ligation Assay

To evaluate the presence and amount of AON in tissues, an AON sequence-specific hybridization ligation assay was performed. We analyzed the diaphragm from treated and untreated mice given that the diaphragm from alginate-ZM2-AON treated mice resulted positive in ESRA analysis (8% of skipping). To compare the data with a muscle that was negative in the ESRA analysis, we also analyzed the quadriceps from untreated and treated mice. The AON hybridization assay revealed the absence of AON both in diaphragm and quadriceps from all the treatments, probably due to the low amount of AON administered in our experiments (240 mg/kg) and M23D could be too low to be detectable by this assay (data not shown).

#### 2.2.4. Immunofluorescence Analysis

In order to evaluate the presence and the correct localization of dystrophin, wild type (WT), treated and untreated *mdx* sections were double labeled with a polyclonal antidystrophin antibody, followed by an antilaminin alpha2 chain antibody for skeletal and cardiac muscles [36] and an antidesmin antibody for the intestine.

**Figure 4:**
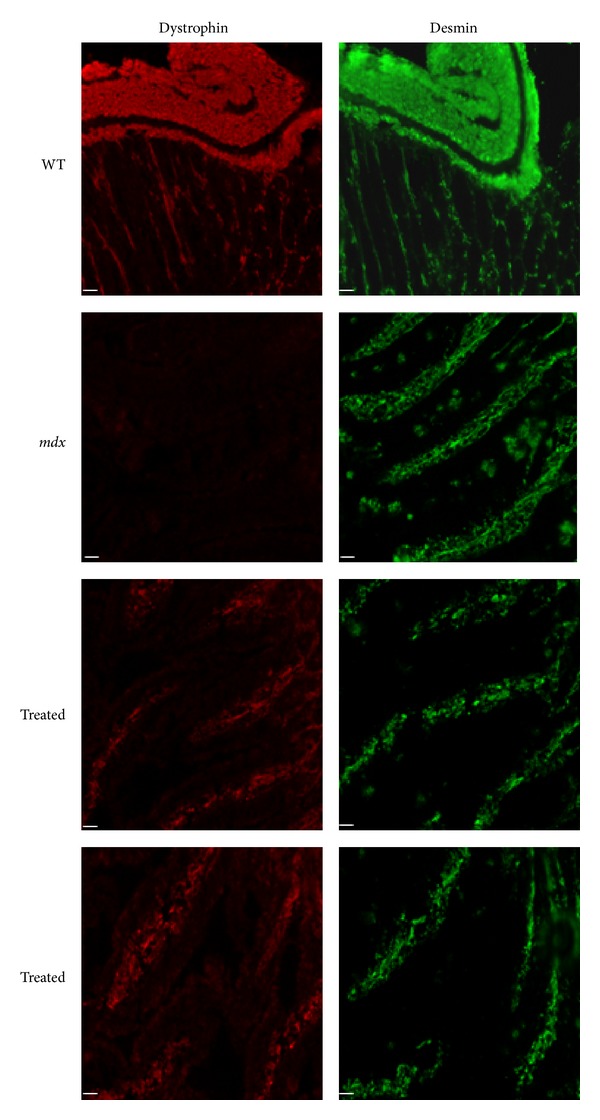
Immunofluorescence analysis of intestinal smooth muscle of wild type (WT), untreated (*mdx*) and alginate-ZM2-M23D orally treated (Treated) *mdx* mice. The sections of small intestine were labeled with antidystrophin antibody. Serial sections of intestine were labeled with a polyclonal antibody for desmin (green). All samples were observed with a Nikon Eclipse 80i fluorescence microscope. Dystrophin (red) is clearly visible in the intestinal smooth muscle of WT mice, absent in untreated *mdx*, and rescued in treated *mdx* mice. (Scale bar = 50 *μ*m).

**Figure 5:**
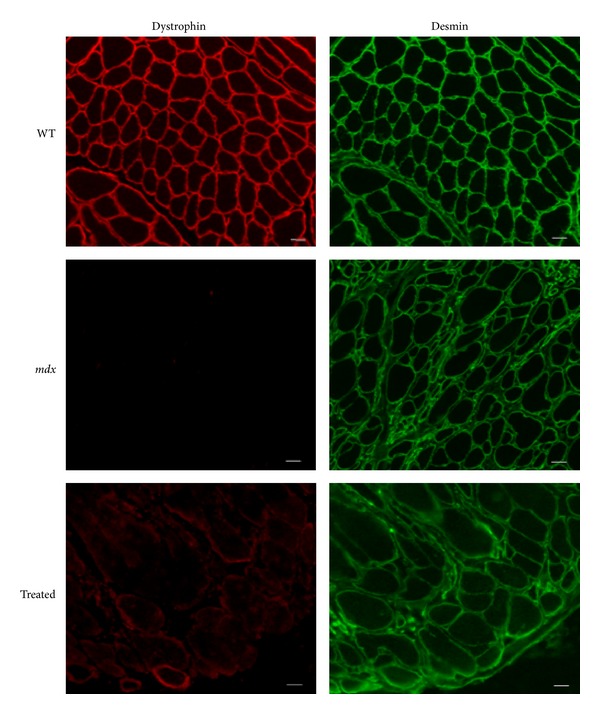
Immunofluorescence analysis of dystrophin protein in the diaphragm of wild type (WT), untreated (*mdx*), and alginate-ZM2-M23D orally treated (treated) *mdx* mice. The sections were labeled with antidystrophin antibody (red signal) and double-labeled with a rat monoclonal antibody for laminin-2 (green). Dystrophin traces are visible in diaphragm from alginate ZM2-M23D oral treated mice. No dystrophin labeling was detected at the sarcolemma in mice untreated or treated with ZM2-AON *mdx*. (Scale bar = 50 *μ*m).

After 12 weeks of treatment with alginate ZM2-AON, immunofluorescence analysis revealed a slight rescue of dystrophin only in intestinal smooth muscle (Figure 4) and diaphragm (Figure 5); the latter result being consistent with the RNA data.

In mice treated with ZM2-AON alginate-free complexes, no dystrophin was detected in any of the muscles analyzed.

#### 2.2.5. Western Blotting

**Figure 6:**
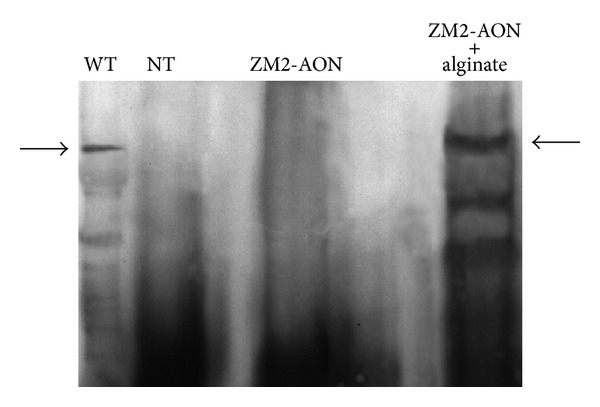
Immunoblotting of dystrophin in the intestine of untreated *mdx* mice (NT), ZM2-AON treated mice, and alginate-coated ZM2-AON treated mice. Intestine from WT mice were used as positive controls. For WT samples, the total protein loaded was 1/10 (15 *μ*g) of the quantity used in the other lanes (150 *μ*g). Quantitation performed by densitometric analysis of autoradiographic bands followed by normalization with the quantity of total protein loaded on the gels showed 31 ± 1.5% (*P* = 0.0003) of dystrophin recovery in the intestine from alginate ZM2-AON-treated mice compared to WT mice. The arrows show the bands of dystrophin (427 kD).

Western blot analysis, performed as previously described [13], confirmed the presence of high molecular-weight dystrophin protein only in the intestine smooth muscle from alginate ZM2-AON-treated mice (Figure 6).

For WT samples, the total protein loaded was 1/10 (15 *μ*g) of the quantity in the other lanes (150 *μ*g). Quantitation performed by densitometric analysis of autoradiographic bands followed by normalization with the quantity of total protein loaded on the gels showed 31 ± 1.5% (*P* = 0.0003) of dystrophin recovery in the intestine from alginate ZM2-AON treated mice with respect to WT mice controls. The results are representative of three separate experiments. Data are given as means ± S.E.M.; statistical significance was calculated with the Student's *t*-test for unpaired data. No protein was detected in skeletal muscles (gastrocnemius, quadriceps, and diaphragm) or the heart of alginate ZM2-AON treated mice. No dystrophin was detected in tissues/muscles from all the other treated mice analyzed. The high (31%) percentage of dystrophin in the smooth muscle of intestine, together with the positivity immunostaining, might be not surprising considering the oral administration route.

## 3. Discussion

In this work, we demonstrate that the oral route is very appealing as administration for AON and, more in general, drugs. The promising fact is the very low, but measurable, dystrophin rescue on the diaphragm that needs to be further studied to improve efficiency, reaching a remarkable dystrophin restoration. We believed that our data, although preliminary, might represent the first step for further studies aiming at delivering nanoparticle-AON orally.

Here, we reveal the biodistribution and elimination of orally administered NIR-dye marked NPs as well as the exon skipping efficacy of NPs combined with AON molecules in *mdx* mice.

Preclinical studies and clinical trials [1, 4, 5, 7, 37] have described the use of AONs limited to parenteral, intraperitoneal (i.p.), intravenous (i.v.), and subcutaneous (s.c.) routes [13–15, 19, 38–40]. To date, the oral route has received limited attention because it is known that AON-RNA molecules are subjected to the depurination process in the acidic gastric environment [22–25].

Here, we use alginate as an encapsulating agent for the oral formulation in order to protect the AON further, in addition to its combination with nanoparticles, against the gastric pH and to increase the intestinal adhesiveness of ZM2-AON complexes. Sodium alginate is biocompatible, biodegradable, nontoxic and approved by the US Food and Drug Administration for oral use [41–46]. It is also extensively used in food industry as a thickener, emulsifier and as a stabilizer [33]. Until now, alginates have been used for oral administration in preclinical models to encapsulate antitubercular drugs [43], hepatitis B antigen [47], pDNA coding for fish lymphocystis disease virus (LCDV) [48], insulin [49], the anti-inflammatory tripeptide Lys-Pro-Val (KPV) to treat inflammatory bowel disease (IBD) [50], oral DNA vaccine against infectious pancreatic necrosis virus [51], and antisense DNA oligonucleotides [46].

Alginate has never been used as a protective agent for exon-skipping inducing RNA molecules systemic delivery.

After a single oral administration, ZM4 remains in the intestine for about 72 hours, before being adsorbed and/or eliminated. The imaging in live animals before each dose of the multiple treatment shows the absence of NP accumulation. Adsorption through the intestinal wall is demonstrated by Odyssey cryosection analysis showing ZM4 in abdominal lymph nodes one week after treatment; no ZM4 was detected in the organs of mice sacrificed 1 month after the last administration. ZM4 clearance studies reveal that they are eliminated almost exclusively through feces and mainly in the first 48 hours after administration. This result suggests that the positivity observed in the intestine 72 hours after administration is represented by a low percentage (10%) of residual ZM4 and that the biodistribution evaluation could be affected by this early elimination of the majority of the administered NPs. Furthermore, in this context, the potential functional effect of NP-AON complexes in terms of dystrophin protein rescue could be reduced by the low amount of systemically available therapeutic molecules.

Following the verification of the intestinal absorption of the ZM4 we continued to demonstrate that oral treatment with NP-AON complexes is able to restore dystrophin synthesis, though at very low level, in *mdx* mice. The comparison between two different treatments, one with ZM2-AON complexes encapsulated with alginate and the other with alginate-free ZM2-AON complexes, reveals that the alginate is necessary to protect the AON molecules, maintaining their ability to recover the dystrophin protein.

Our data demonstrate that only in *mdx* mice orally treated with alginate-coated ZM2-AON complexes there is a slight rescue of dystrophin protein in the intestinal smooth muscles and a weak positivity in the diaphragm. No dystrophin was detected, on the other hand, in any of the muscles of mice treated with alginate-free ZM2-AON complexes.  Table 4 summarizes the dystrophin restoration results.

It is known that dystrophin is present in several cell types in the gastrointestinal wall, in smooth muscle cells (SMCs) of the muscularis externa and muscularis mucosae, in the myoid cells located in the mucosa, in the perivascular SMCs, and all the submucous and myenteric neurons [52]. On the contrary, full-length dystrophin is lacking in the gastrointestinal tract of DMD patients and *mdx* mice, which show several alterations in gastrointestinal motility.

Notably, our results give the first evidence that the oral delivery of an antisense oligoribonucleotide co-formulated with ZM2 and alginate can produce a functional effect on RNA splicing. We observe that the protective action of the alginate is essential for preventing damage to the AON molecules by gastric acid since only with alginate-coated ZM2-AON we observe dystrophin synthesis, showing that ZM2 alone are not sufficient for AON protection. The low efficiency on the restoration of dystrophin protein might be due to the inability of ZM2 to efficiently overcome the intestinal barrier, as documented by the biodistribution studies by Odyssey. Concomitantly, the observed laxative effect of alginate may result in a reduced amount of available ZM2-AON complexes and a consequent decrease in the contact time between particles and the intestinal epithelium, leading to a lower uptake of the molecules. AON specific ELISA concordantly demonstrated the absence of AON in mice tissues. Nonetheless, some AONs are protected enough to pass the body barriers and reach at least the diaphragm with measurable functional effect.

Smaller size or/and modified hydrophilicity of NPs and AON chemical modifications may also improve the *in vivo* biodistribution and stability of the binding with NPs-AON and therefore deserve to be addressed. In conclusion, the challenge for future studies will be to improve both the AON structure (backbone) and molecule protection by NPs to improve the AON bioavailability and to enhance the intestinal cells' uptake of orally delivered drugs.

## 4. Materials and Methods

### 4.1. Animals

All experiments were performed on male *mdx* mice (C57BL/10ScSn-Dmd*mdx*/J). All procedures were approved by the Animal Experimentation Ethics Committee. Mice were housed in temperature-controlled rooms (22°C) at a humidity level of 50% and a 12:12 hour light-dark cycle. Mice were purchased from the Jackson Laboratory (Bar Harbor, ME).

### 4.2. ZM4 Nanoparticles Synthesis

ZM4 nanoparticle sample were prepared by emulsion polymerization of methyl methacrylate employing as emulsion stabilizers, different functional comonomers (N-isopropyl acrylamide (NIPAM), (N,N-dimethyl N-octyl ammonium) ethyl methacrylate bromide (MOAEMA) and 2-aminoethyl methacrylate hydrochloride (AEMA)).

In particular, 30.0 mL of methyl methacrylate were introduced in a flask containing 500 mL of an aqueous solution of 2.34 g (6.7 mmol) of MOAEMA, 0.55 g (3.3 mmol) of AEMA and 1.13 g (10 mmol) NIPAM. The flask was fluxed with nitrogen under constant stirring for 30 minutes, then 85 mg (0.313 mmol) of the cationic free radical initiator AIBA, dissolved in water, were added. The polymerization was performed at 80 ± 1.0°C for 4 hours under constant stirring. At the end of the reaction, the samples were recovered and purified by repeated dialysis.

Particle sizes and size distributions were measured by dynamic light scattering and scanning electron microscopy (SEM) analysis. Dynamic light scattering analysis was performed at 25°C, with a Malvern Zetasizer Nano ZS system at a fixed scattering angle of 90°, using a He-Ne laser and a PCS software (Malvern, U.K., version 6.11). Five individual measurements were performed for each sample. The values that we report are the average of these 5 measurements. SEM analysis were performed with a Field Emission Gun Inspect F microscope from FEI Company. The SEM micrographs were elaborated by the Scion Image processing (NIH, public domain) program. From 200 to 250 individual nanospheres were measured for each sample. The characteristics of the obtained nanoparticles are reported in Table 1.

### 4.3. ZM4 Conjugation with NIR-797 Dye

1,2 mL of a solution 3 mg/mL of dye NIR-797 Isothiocyanate (Sigma-Aldrich, *λ*_ex_ 795 nm; *λ*_em_ 817 nm) in anhydrous DMSO were added to 15 mL of a suspension of nanoparticles (22 *μ*g NIR-797/mg nanoparticle) in phosphate buffer (10 mM, pH 8) under magnetic stirring for 2 hours protected from light. After centrifugation (10 minutes at 11000 g) the pellet was washed twice, then resuspended in water obtaining ZM4-IR nanoparticles.

### 4.4. Oral Administration Schedule for Biodistribution and Elimination Studies

2.5 mg of ZM4-IR NPs dissolved in 200 *μ*L of water, were administered to mice by oral gavage in single or multiple treatments. 8–6 weeks old *mdx* mice were divided into 2 groups (*n* = 2) of treatment: the first group received a single dose of ZM4-IR, the second group received 2 administrations/week for a total of 15 administrations of ZM4-IR. Table 2 summarizes the administration schedule. Two untreated mice were used as negative controls.

### 4.5. Biodistribution and Elimination Studies

Prior to the acquisition of *in vivo* images, the mice were fed with a special diet (Research Diets AIN-76A or AIN-93G) in order to minimize autofluorescence (red signal) due to chlorophyll contained in plant-based ingredients. The animals were anesthetized with 1% Avertin, shaved in abdominal and chest regions and located on MousePod of Odyssey for infrared *in vivo* imaging. The scanning was performed 12, 24, 48, 72 hours after a single administration of ZM4-IR and before each dose for multiple administrations. For nanoparticles clearance studies mice were placed into metabolic cages for collecting feces and urine for 24 hours starting after 12 hours and 6 days after ZM4-IR administration. The feces were weighed and then dissolved in 4 mL of PBS by vigorous shaking, while the urine samples were spin for 10 minutes at 11000 g and solubilized in 100 *μ*L. The total urine sample (100 *μ*L), 100 *μ*L of feces suspension and progressive dilutions of both samples were put in a 96 well-dish and analyzed by Odyssey. Along with samples a dilution of the fluorescent nanoparticles were prepared as a standard. To perform quantification of signals, regions of interest (ROIs) of equal size were drawn and placed over each well. A calibration curve was obtained from the dilution of the fluorescent nanoparticles after normalization of each ROI to the sum of the total intensity of all the standard ROIs. The same normalization procedure was applied to the feces and urine samples and normalized values were used.

At the end of treatments, mice were sacrificed, their tissues and organs excised, frozen in liquid N2-cooled isopentane, and stored at −80°C. Twenty-micrometer-thick frozen transverse sections were cut from different specimens, analyzed by Odyssey and stained with hematoxylin and eosin (H&E) (Sigma-Aldrich, Milan, Italy).

### 4.6. AON Synthesis

M23D(+07-18) (5′-GGCCAAACCUCGGCUUACCUGAAAU-3′) AON against the boundary sequences of the exon and intron 23 of mouse *dystrophin* gene, contains 2′-O-methyl modified RNA and full-length phosphorothioate backbone. Oligonucleotide synthesis was carried out on an ÄKTA oligopilot plus 10 DNA/RNA synthesizer (GE Healthcare, Milano, Italy) using its trityl-on mode. The sequence was synthesized on a 2 *μ*mol scale using Primer Support 200 loaded at 80 *μ*mol/g (Amersham Biosciences, Milano, Italy). Commercial 2′-O-methyl phosphoramidites (Proligo, Boulder, CO) were dissolved to a nominal concentration of 50 mmol/L in anhydrous acetonitrile (CH_3_CN) and activated with a 0.25 mol/L solution of 5-(bis-3,5-trifluoromethylphenyl)-1H-tetrazole (Proligo) in CH_3_CN. The final detritylation was achieved using a 0.1 mol/L aqueous solution of NaOAc (pH 3.0). Crude dimethyltryptamine protected and detritylated oligonucleotide were purified by an ÄKTAbasic ultraphysical contact liquid chromatography system using an Amersham Biosciences Resource RPC 3 mL column eluted under a gradient of CH_3_CN in 0.1 mol/L triethylammonium acetate (pH 8). The final oligonucleotide was dissolved in water and filtered through a short column of Dowex 50WX8 (Na^+^ form, 100–200 mesh) to obtain after lyophilization 0.8 *μ*mol (40%) of target compound. The purity of the full-length desired product was evaluated by MALDI-TOF MS, 31P-NMR and RP-HPLC analyses.

### 4.7. ZM2-M23D Loading

ZM2 nanoparticles were prepared by emulsion polymerization of methyl methacrylate employing, as emulsion stabilizers, two functional comonomers (N-isopropyl acrylamide (NIPAM) and (N,N-dimethyl N-octyl ammonium) ethyl methacrylate bromide) as previously reported [14]. They have a particle diameter of 137 nm (as determined by scanning electron microscopy) and a surface charge density of 202 *μ*mol ammonium groups per gram of nanoparticles. M23D AON contains 2′-O-methyl RNA phosphorothioate backbone and its synthesis was previously described [13]. Loading experiments have shown that ZM2 nanoparticles (1 mg/mL) adsorbed 2′OMePS M23D oligoribonucleotide onto their surface in the concentration range of 10–100 *μ*g/mL. M23D adsorption onto ZM2 nanoparticles is a highly reproducible process with a loading value of 90 *μ*g/mg [14].

### 4.8. Naked ZM2 or ZM2-AON Coating with Alginate

Suspension of naked ZM2 or ZM2-AON complexes in water were mixed with a solution of sodium alginate (0.52 mg alginate/mg of ZM2) under magnetic stirring for 10 minutes. CaCl_2_ (3 mM) was added to the solution and kept under agitation for 10 additional minutes. After centrifugation at 11000 g for 15 minutes the supernatant was removed and collected in new tubes, while alginate-ZM2 or alginate ZM2-AON (200 *μ*g AON/2.5 mg ZM2) pellets were loaded in insulin syringes for oral administration by gavage. The collected supernatants were drawn, filtered on a Millex GV_4_ filter and the absorbance (260–280 nm) measured by Nanodrop 1000 (Thermo Scientific).

### 4.9. Oral Administration Schedule for Dystrophin Restoration Studies

Two groups of *mdx* mice (6 weeks old) were treated by oral gavage. The I group received 200 *μ*L of suspension containing 225 *μ*g of M23D loaded onto 2.5 mg of ZM2 nanoparticles (3 mice) or 2.5 mg of AON-free ZM2 nanoparticles as negative control (3 mice) for a total of 32 administrations (240 mg/Kg total). The II group was treated with 200 *μ*L of suspension containing 200 *μ*g of M23D loaded onto 2.5 mg of ZM2 nanoparticles or 2.5 mg of AON-free ZM2 nanoparticles but in the presence of alginate (3 mice for each different treatment) for a total of 36 administrations (240 mg/Kg total). Three untreated age-matched *mdx* mice were included as negative controls. All groups of mice were sacrificed 1 week after last administration.

Table 3 summarizes the administration schedule.

### 4.10. Dystrophin RNA Studies

Total RNA was extracted from frozen sections of muscle biopsies using TRIzol (Invitrogen, Milano, Italy), and reverse-transcribed into cDNA using the High-Capacity cDNA Reverse Transcription kit (Applied Biosystems, Frankfurt, Germany). We performed Exon-Specific Real Time Assays (ESRAs) using RNA pool for each treatment group. ESRAs were used on exons 8, 23, and 25 to quantify the percentage of exon-23 skipping in treated, with respect to untreated mice (ΔCt method), using *β*-actin as endogenous control. All these ESRAs are based on TaqMan technology and have been designed by PrimerExpress Applied Biosystems software (Applied Biosystems). Primers and probes sequences are available upon request. The amount of target sequences compared to appropriate endogenous control (*β*-actin gene) was evaluated by the comparative CT method with respect to the endogenous *β*-actin control (ΔΔCt Method) (Applied Biosystems User Bulletin no. 2) [13, 53].

### 4.11. AON Hybridization Ligation Assay

The assay for measuring the concentration of 2OMePS oligoribonucleotide 23AON in tissue samples is based on a hybridization ligation assay [38]. A template probe (5′-gaatagacgaggtaagccgaggtttggcc-biotin-3′, 29-mer DNA phosphate oligonucleotide) and a ligation probe (5′-P-cgtctattc-DIG-3′, 9-mer DNA phosphate oligonucleotide) were used. The sample was incubated with the template probe (50 nmol/L) at 37°C for 1 hour, and the hybridized samples were transferred to streptavidin-coated 96-well plates. Subsequently, the digoxigenin-labeled ligation probe (2 nmol/L) was added. Detection was performed with anti-DIG-POD (1 : 4,000; Roche Diagnostics, Milan, Italy), 3,3′,5,5′-tetramethylbenzidine substrate (Sigma Aldrich S.r.l, Milan, Italy), and stop solution (Sigma Aldrich). Absorption at 450 nm was measured in a SpectraFluorPlus (Tecan Italia Srl, Cernusco sul Naviglio, Milan, Italy). The tissue samples were homogenized to a concentration of 60 mg/mL in proteinase K buffer (100 mmol/L Tris-HCl, pH 8.5, 200 mmol/L NaCl, 5 mmol/L EDTA, 0.2% sodium dodecylsulfate) containing 2 mg/mL of proteinase K (Life Technologies, Monza, Italy), followed by incubation for 4 hours rotating at 55°C in a hybridization oven. Next, the samples were centrifuged for 15 minutes at maximum speed and the supernatant was used in the assay. We analyzed diaphragm and quadriceps from untreated and oral treated *mdx* mice. Muscle samples were first diluted 60-fold in PBS, subsequent dilutions and the calibration curves were done in 60-fold control muscle in PBS.

### 4.12. Dystrophin Protein Immunofluorescence Analysis

One week after the last injection, *mdx* mice were killed, and their diaphragm, quadriceps, gastrocnemius, cardiac muscles and intestine isolated, snap-frozen in liquid N2-cooled isopentane and stored at −80°C until further processing.

Seven-micrometer-thick frozen transverse sections were cut from at least two-thirds of the length of heart, diaphragm, gastrocnemius, quadriceps muscles and intestine; for each muscle at least 5 slices were cut at 150 *μ*m intervals. To analyze dystrophin restoration freshly-cut muscle and intestine sections were labeled with a polyclonal antidystrophin antibody (H-300) (epitope corresponding to amino acids 801–1100 mapping within an internal region of dystrophin; Santa Cruz Biotechnology, Santa Cruz, CA) diluted 1 : 100 revealed with anti-rabbit Cy3-conjugated secondary antibody (Jackson Immunoresearch, Suffolk, UK). Muscles samples were double-labeled with a rat monoclonal antibody for laminin-2 (*α*2-chain, 4H8-2, diluted 1 : 500; Alexis Biochemical, Farmingdale, NY, USA) revealed with anti-rat Cy2-conjugated secondary antibody (Jackson Immunoresearch, Suffolk, UK). Seriate sections of intestine were labeled with a polyclonal antibody for desmin (diluted 1 : 100, Abcam, Cambridge, UK) followed by anti-rabbit FITC-conjugated secondary antibody for desmin (DAKO, Glostrup, Denmark). All images were observed with a Nikon Eclipse 80i fluorescence microscope (Nikon Instruments, Firenze, Italy) connected to at a high-resolution CCD camera (Nikon Instruments, Firenze, Italy) at 20x magnification.

### 4.13. Dystrophin Analysis by Western Blotting

Western blot analysis was performed as previously described [13]. Twenty-micrometer-thick frozen muscle sections were homogenized with a lysis buffer (7 mol/L urea, 2 mol/L thiourea, 1% amidosulfobetaine-14, and 0.3% dithioerythritol). Aliquots of proteins from wild type mice (15 *μ*g) and from the muscles of treated or untreated *mdx* mice (150 *μ*g) were loaded onto a 6% sodium dodecyl sulphate-polyacrylamide gel and separated by electrophoresis. Samples were transferred to a nitrocellulose membrane at 75 V. The membrane was incubated overnight at 4°C with the specific antibody DYS2 (NovoCastra, Newcastle, UK).

To quantify the restoration of dystrophin protein in treated versus wild type mice, a densitometric analysis of autoradiographic bands was performed with a Bio-Rad Densitometer GS 700 (Bio-Rad, Milan, Italy), followed by normalization with the quantity of total protein loaded onto the gels.

## Figures and Tables

**Table 1:** Nanoparticles characteristics.

Sample	Diameter (SEM) nm (±standard error of the mean)	Diameter (PCS) nm (±polydispersity index)	Quaternary ammonium groups *μ*mol/g	Primary amino groups *μ*mol/g	NIPAM	Studies
ZM2	137 ± 9	156 ± 0.015	202	—	yes	Functional analysis (dystrophin rescue) with ZM2-AON and ZM2-AON alginate

ZM4	138 ± 7	143 ± 0.012	107	10.2	yes	Biodistribution and elimination using dye NIR-797

**Table 2:** Schedule of IR-dye conjugate nanoparticle treatments for biodistribution and elimination analysis.

No. ofmice	Oral formulations	Frequency	No. of administration	Time of analysis
*n* = 3	ZM4-IR (2.5 mg)	1/week	1	Elimination (metabolic cages): 12 hours after administration Time of collection of Odyssey images: 12–24–48–72 hours

*n* = 3	ZM4-IR (2.5 mg)	2/week	15	Time of collection of Odyssey images: before the new administration Sacrifice: 1 week, 1 month after last administration

**Table 3:** Schedule of *mdx* treatments for dystrophin restoration studies.

	Oral formulations	Frequency	No. of administrations	Total dose	Time of analysis
Group 1 Alginate-free (*n* = 6)	ZM2 (2.5 mg)-AON (225 μg) (*n* = 3)	2/week	32	240 mg/Kg	1 week after last administration
	ZM2 (2.5 mg) (*n* = 3)	2/week	32	—	1 week after last administration

Group II Alginate-coated (*n* = 6)	ZM2 (2.5 mg)-AON (200 μg) (*n* = 3)	3/week	36	240 mg/Kg	1 week after last administration
	ZM2 (2.5 mg) (*n* = 3)	3/week	36	—	1 week after last administration

Group III Untreated (*n* = 3)	—	—	—	—	1 week after last administration

**Table 4:** Summary of results of dystrophin restoration studies.

	Oral formulations	Tissues	Exon 23 skipping %	IHC	WB
Group I No alginate (*n* = 6)	ZM2 (2.5 mg)-AON (225 μg) (*n* = 3)	Diaphragm	0	−	−
		Intestine	0	−	−
		Gastrocnemius	0	−	−
		Quadriceps	0	−	−
		Heart	0	−	−
	ZM2 (2.5 mg) (*n* = 3)	Diaphragm	0	−	−
		Intestine	0	−	−
		Gastrocnemius	0	−	−
		Quadriceps	0	−	−
		Heart	0	−	−

Group II +alginate (*n* = 6)	ZM2 (2.5 mg)-AON (200 μg) (*n* = 3)	Diaphragm	8	+	−
		Intestine	0	+	+
		Gastrocnemius	0	−	−
		Quadriceps	0	−	−
		Heart	0	−	−
	ZM2 (2.5 mg) (*n* = 3)	Diaphragm	0	−	−
		Intestine	0	−	−
		Gastrocnemius	0	−	−
		Quadriceps	0	−	−
		Heart	0	−	−

Group III Untreated (*n* = 3)	—	Diaphragm	0	−	−
		Intestine	0	−	−
		Gastrocnemius	0	−	−
		Quadriceps	0	−	−
		Heart	0	−	−
